# P-FN12, an H4R-Based Epitope Vaccine Screened by Phage Display, Regulates the Th1/Th2 Balance in Rat Allergic Rhinitis

**DOI:** 10.1016/j.omtm.2018.09.004

**Published:** 2018-10-04

**Authors:** Yuqian Wang, Jichao Sha, Heng Wang, Lifeng An, Tie Liu, Lin Li

**Affiliations:** 1 Scientific Research Center, China-Japan Union Hospital of Jilin University, Changchun, Jilin 130033, China; 2 Department of Otorhinolaryngology Head and Neck Surgery, China-Japan Union Hospital of Jilin University, Changchun, Jilin 130033, China; 3 Environmental Protection Research Institute of Changchun, 7930 Weixing Road, Changchun 130022, China; 4 Biobank, China-Japan Union Hospital of Jilin University, 126 Xiantai Street, Changchun 130031, China

**Keywords:** allergic rhinitis, histamine receptor 4, Th1/Th2 responses, phage display peptide library, epitope

## Abstract

Allergic rhinitis (AR) involves antigen-specific immune-inflammation of the nasal mucosa. Classical therapy for AR targets the histamine pathway, e.g., histamine receptor blockers. Histamine H4 receptor (H4R) was suggested as a novel therapeutic target due to its wide expression on almost all immune-related cells. A 12-mer random peptide library was used to select the specific epitope of the H4R. The phage clone showing the highest degree of activation was verified and translated to the corresponding peptide. The peptide FNKWMDCLSVTH, designated as P-FN12, was bound by H4R monoclonal antibody (mcAb) with high affinity. Moreover, the P-FN12 + CTB@Lipo-formulated vaccine, used as nasal drops, decreased allergic symptoms such as sneezing and nasal rubbing in a rat model. The level of ovalbumin (OVA)-specific immunoglobulin E (IgE) decreased significantly after vaccine administration. It also elicited increased levels of interferon (IFN)-γ and interleukin-2 (IL-2) but a decreased level of IL-4, and it elevated the T helper type 1 (Th1):T helper type 2 (Th2) cell ratio in peripheral blood mononuclear cell cultures. Our results indicated that the reduction of allergic inflammation by P-FN12-based vaccine was related to a decrease in production of OVA-specific IgE, Th2 immunity, and tissue eosinophilia. P-FN12 + CTB@Lipo is a promising vaccine that could suppress Th2 response and enhance the induction of Th1 cells in an AR model.

## Introduction

Allergic rhinitis (AR) is a significant health issue affecting approximately 500 million people worldwide.^1^ AR is regarded as an immunological disorder caused by genetic and environmental factors. Moreover, AR is triggered by inflammation of nasal mucosa with hypersensitivity resulting from various types of allergens. It is widely accepted that sensitization is induced by a dominance of T helper type 2 (Th2) cell activation and an inadequate T helper type 1 (Th1) cell response, with subsequent allergic inflammation in response to invading allergens. Improving the Th1/Th2 balance is considered an effective means of relieving the symptoms of AR.^2^

Histamine has long been recognized as an important mediator of allergic inflammation.^3^ To date, four subtypes of histamine receptor, H1, H2, H3, and H4, have been described. The histamine H4 receptor (H4R) was identified most recently, and it was shown to be involved in the recruitment and activation of cells involved in allergic inflammatory responses, including eosinophils, T cells, dendritic cells, basophils, and mast cells.^4^, ^5^ It is demonstrated that the H4R modulates inflammation in a chronic allergic dermatitis setting; thus, it is necessary to block H4R during ontogeny and development of the allergic inflammation.^6^, ^7^ It has been demonstrated that blocking both H1R and H4R has additive effects in preventing the intestinal consequences of peanut sensitization and challenge.^8^ Moreover, pharmacological studies suggest the potential utility of histamine H4 antagonists in the treatment of inflammatory diseases, such as AR, asthma, atopic dermatitis, and pruritus.^9^, ^10^, ^11^, ^12^, ^13^ The selective H4R antagonist JNJ7777120 showed efficacy in relieving symptoms and inflammatory conditions in animal models of AR.^11^, ^14^ However, these treatments are not curative and they are expensive; additionally, antihistamines may impair performance due to their side effects.^15^, ^16^

Specific immunotherapy (SIT) is considered the only disease-modifying treatment for AR, considering its ability to alter the Th2-biased immune response, while pharmacotherapy acts only on symptoms.^17^ Antigen-specific immunotherapy can alter the natural course of AR, and it is recognized as a curative treatment for type I allergy without risks of impaired performance. H4R is expressed in many immune cells, such as eosinophils, T cells, dendritic cells, basophils, and mast cells. Immunotherapy targeting H4R can comprehensively improve the immune system, and it is not limited to any specific antigen due to the extensive expression.

In this study, a peptide mimic H4R was discovered, and its immunogenicity and Th1/Th2 immune response imbalance correction in an AR rat model were examined. Overlapping synthetic peptides and phage display libraries were used to identify the potential epitopes of H4R recognized by anti-H4R monoclonal antibody (mcAb). An epitope of H4R (peptide sequence FNKWMDCLSVTH, designated as P-FN12) was identified for its immunogenicity in a rat AR model. The specificity of the identified epitope (P-FN12) was evaluated by ELISA. P-FN12 was combined with cholera toxin B (CTB) and liposomes (Lipofectamine; Invitrogen, Carlsbad, CA) to verify its immunogenicity. The cytokine levels in peptide- and adjuvant-immunized rats were determined in the serum and nasal mucosa, and the Th1:Th2 lymphocyte ratio was characterized in peripheral blood mononuclear cells (PBMCs). The results of ELISA showed that P-FN12 bound specifically to anti-H4R antibody, and sera from P-FN12-vaccinated rats indicated high-titer specific antibodies to H4R. The level of ovalbumin (OVA)-specific immunoglobulin E (IgE) increased significantly on intraperitoneal OVA sensitization, but it decreased after vaccination. Immunotherapy downregulated antigen-specific Th2-type responses, but it upregulated Th1-type responses. The number of eosinophils was also decreased in the nasal mucosa after treatment. Our research may facilitate advances in the clinical application of anti-H4R short-peptide vaccines.

## Results

### Identification of Epitopes Using the Phage Display Library and Specificity of Phage P-FN12

In the phage display library, critical amino acid residues within an epitope can be recognized by assessing the sequence of isolated peptides for homology with the primary sequence of the protein antigen. In this study, the nucleotide sequences of 23 positive phage clones were analyzed, and some clones were found to carry the same nucleotide sequence (5′-ACTTTTAAGTTTACGTTGAGTTATCGT CAGGTGCAT-3′). The converted amino acid sequence was FNKWMDCLSVTH, and this peptide was designated as P-FN12.

**Figure 1:**
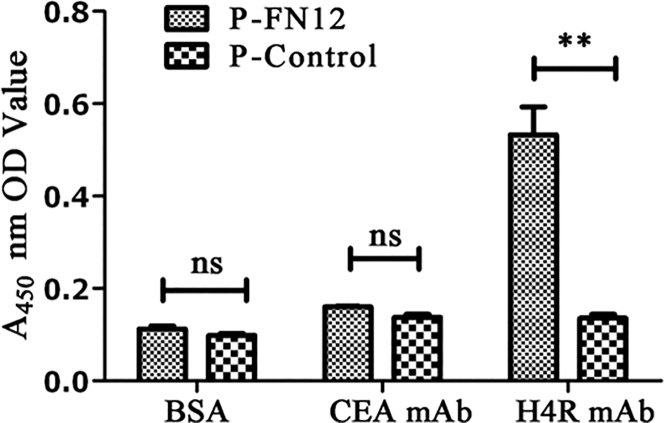
Specificity of Phage P-FN12 as Revealed by ELISA The selected phage peptide P-FN12 reacted with human H4R mcAb, but not human CEA mcAb. The BSA control did not react with either peptide. ns, not significant; **p < 0.01.

The specificity of the identified phage P-FN12 was examined by ELISA. The selected phage P-FN12 was recognized by human H4R mcAb (p < 0.01), but it showed no binding with human carcinoembryonic antigen (CEA) mAb (p > 0.05). Moreover, the BSA control did not bind to either P-FN12 or the P-control (p > 0.05). These results indicated that the selected peptide epitope, P-FN12, bound specifically to anti-H4R antibody (Figure 1).

### H4R-Specific Serum Antibody Levels in Rats Immunized with P-FN12-Based Vaccine

**Figure 2:**
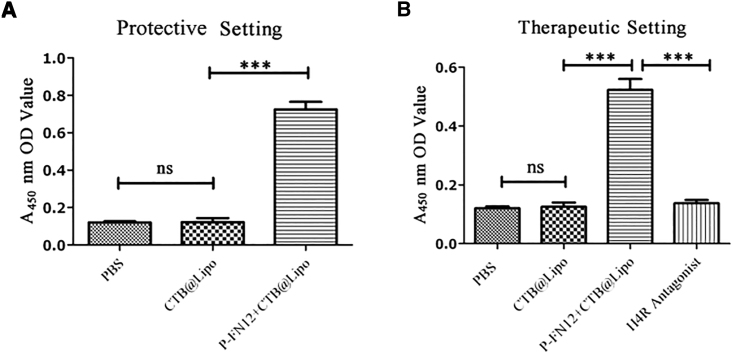
Specificity of Peptide P-FN12 Combined with CTB Formulated with Lipo Antibody levels in pooled serum are shown in the protective setting (A) and therapeutic setting (B) (n = 5). ns, not significant; ***p < 0.001. CTB, cholera toxin B.

The rats were randomly grouped into protective and therapeutic settings (n = 5). The sensitization and immunization procedures were conducted as described in the Materials and Methods. The P-FN12 vaccination group showed approximately 3-fold higher antibody titer than the control groups in both prophylactic and therapeutic settings (p < 0.001) (Figure 2). However, rats in the control group and the CTB@Lipo group did not exhibit detectable serum-specific antibody against H4R at the serum dilutions used in the indirect ELISA, which suggests that P-FN12 + CTB@Lipo could substantially enhance H4R-specific humoral immunity.

### Symptom Score

**Figure 3:**
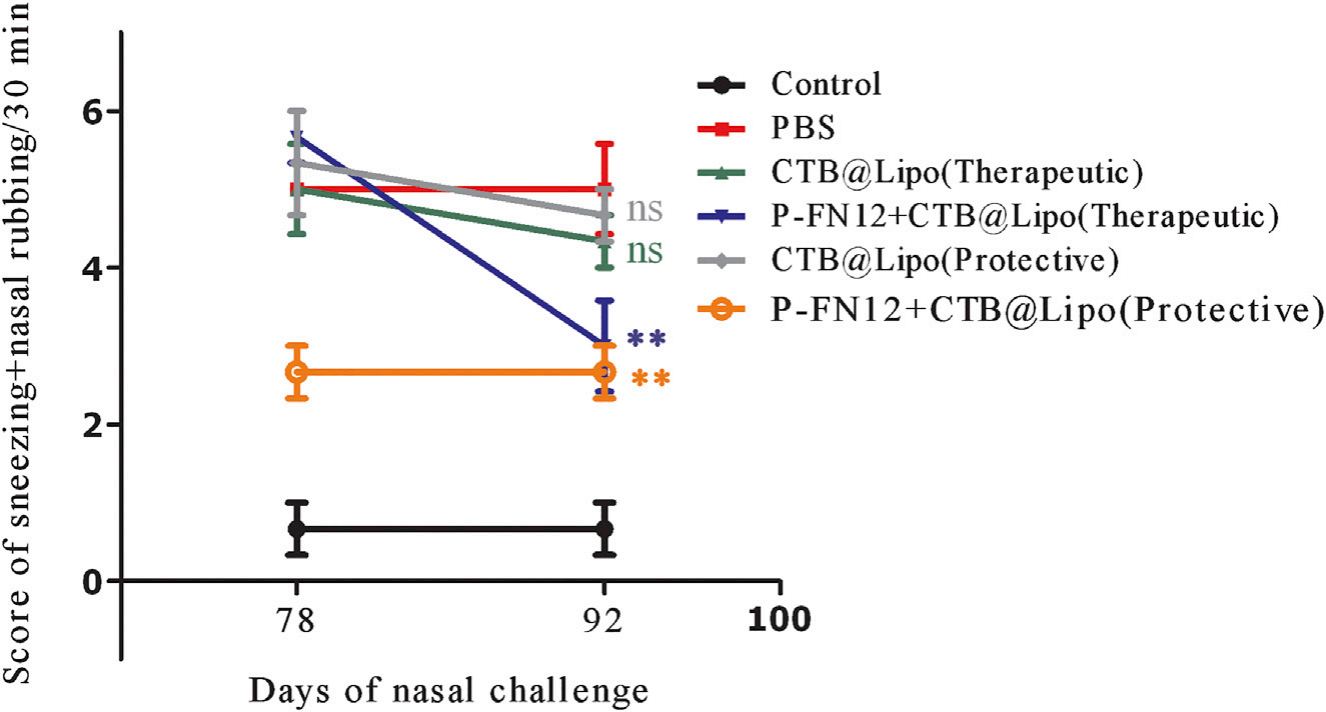
Nasal Symptom Scores Frequency of nasal rubbing plus sneezing on days 78 and 92. The scores shown are the average of the involved mice’s scores in the group (n = 5). ns, not significant; **p < 0.01.

The nasal symptom scores in the P-FN12 + CTB@Lipo group were significantly lower compared with the PBS group, in both the protective and therapeutic settings (p < 0.01) (Figure 3). The nasal symptom score was not significantly lower in the CTB@Lipo treatment group compared to the PBS group (not significant [ns]). All AR groups exhibited higher nasal symptom scores than the control group (no AR). Moreover, the nasal symptom score on day 92 was markedly lower than that on day 78 in the therapeutic setting, whereas there was no significant change in the protective setting. These results indicate that the AR symptoms, such as nasal rubbing and sneezing, were relieved to a large extent in the therapeutic setting. In the protective setting, the symptoms were alleviated before day 72 after sensitization. Thus, the effects of the vaccine may take some time to manifest.

### IgE Detection and Cytokine Expression in Serum and Nasal Mucosa

**Figure 4:**
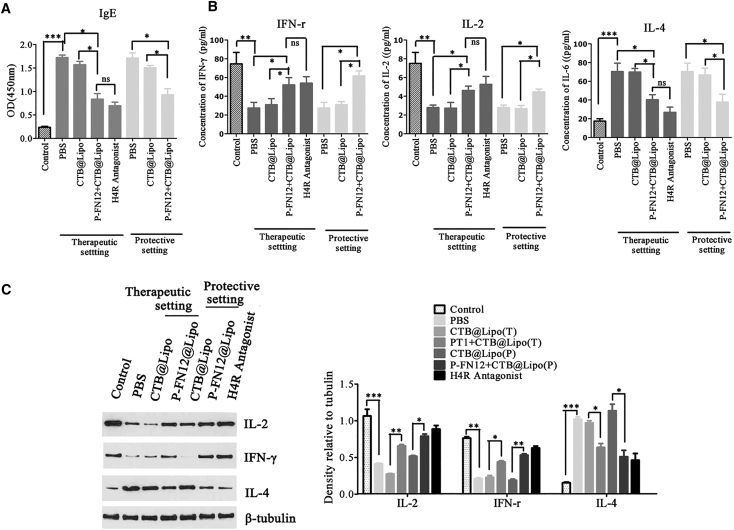
IFN-γ, IL-2, and IL-4 Expression Levels in Serum and Nasal Mucosa (A) OVA-specific IgE levels in serum measured via ELISA. (B) IFN-γ, IL-2, and IL-4 levels in serum as assessed via ELISA in both the therapeutic and protective settings. (C) IFN-γ, IL-2, and IL-4 expression levels in nasal mucosa as revealed by western blotting. The relative densities of the IL-2, IFN-γ, and IL-4 bands were normalized to that of β-actin. Each data point represents the mean ± SD of data from three independent experiments. Control, no AR group; ns, not significant; *p < 0.05, **p < 0.01, ***p < 0.001.

In patients with AR, IgE antibodies are the most common immunoglobulins and the major cause of the inflammatory response. To further examine the therapeutic effects of the P-FN12 + CTB@Lipo vaccine in the AR context, we measured the expression levels of OVA-specific IgE in rat sera via ELISA. Treatment significantly decreased the levels of IgE in both the therapeutic and protective settings (p < 0.05) (Figure 4A), consistent with the observed AR symptom amelioration.

Three different cytokines were measured in the sera and nasal mucosa of vaccinated rats as follows: interleukin-2 (IL-2) and interferon (IFN)-γ, which are specific to the Th1 immune response, and IL-4, which is specific to the Th2 immune response (Figures 4B and 4C). In the protective setting, the expression of the Th2 cytokine IL-4 was significantly lower in the serum (p *<* 0.05), and the expression levels of the Th1 cytokines IFN-γ were significantly higher compared with the PBS group (p *<* 0.05) (Figure 4B). IFN-γ production also increased in the therapeutic setting and IL-4 expression decreased (Figure 4B). Although IL-2 levels did not differ significantly between the PBS and the P-FN12 + CTB@Lipo vaccine groups, the levels tended to align with the tendencies described above.

Cytokine protein expression levels were determined in the nasal mucosa. We examined differences in IFN-γ, IL-2, and IL-4 levels in the therapeutic and prophylactic AR rat groups by western blotting (Figure 4C). As shown, the IL-2 and IFN-γ protein levels in the nasal mucosa of AR rats were significantly increased compared with the PBS group (p < 0.01). Similarly, the IL-4 protein levels were significantly reduced compared with baseline values (p < 0.05).

### Evaluation of Th1/Th2 Cells in PBMCs of Vaccinated Rats

**Figure 5:**
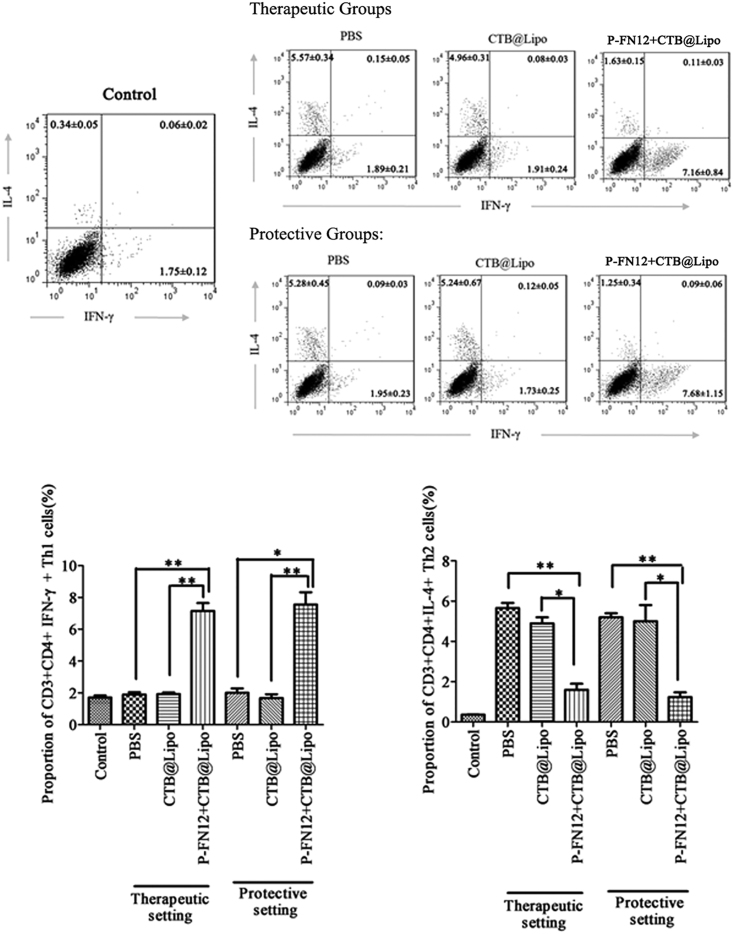
Th1/Th2 Cell Ratios among PBMCs of AR Rats The proportions of CD3^+^CD4^+^ IFN-γ^+^ Th1 cells and CD3^+^CD4^+^IL-4^+^ Th2 cells among PBMCs were determined via flow cytometry (n = 5). PBMCs, peripheral blood mononuclear cells; control, no AR group; *p < 0.05, **p < 0.01.

IFN-γ and IL-4 are key effector cytokines for the differentiation of Th1 and Th2 cells. We examined the proportions of Th1 and Th2 cells in PBMCs by flow cytometry (Figure 5). The control (no AR group) exhibited a normal Th1:Th2 cell ratio. In rats with AR, the P-FN12 + CTB@Lipo-treated group appeared to have a higher percentage of Th1 cells (CD3^+^CD4^+^IFN-γ^+^ cells) in both prophylactic and therapeutic settings. However, the proportion of Th2 cells (CD3^+^CD4^+^IL-4^+^ cells) was lower in the P-FN12 + CTB@Lipo vaccine-treated group than in the PBS group.

### Infiltration of Eosinophils in Nasal Mucosa

**Figure 6:**
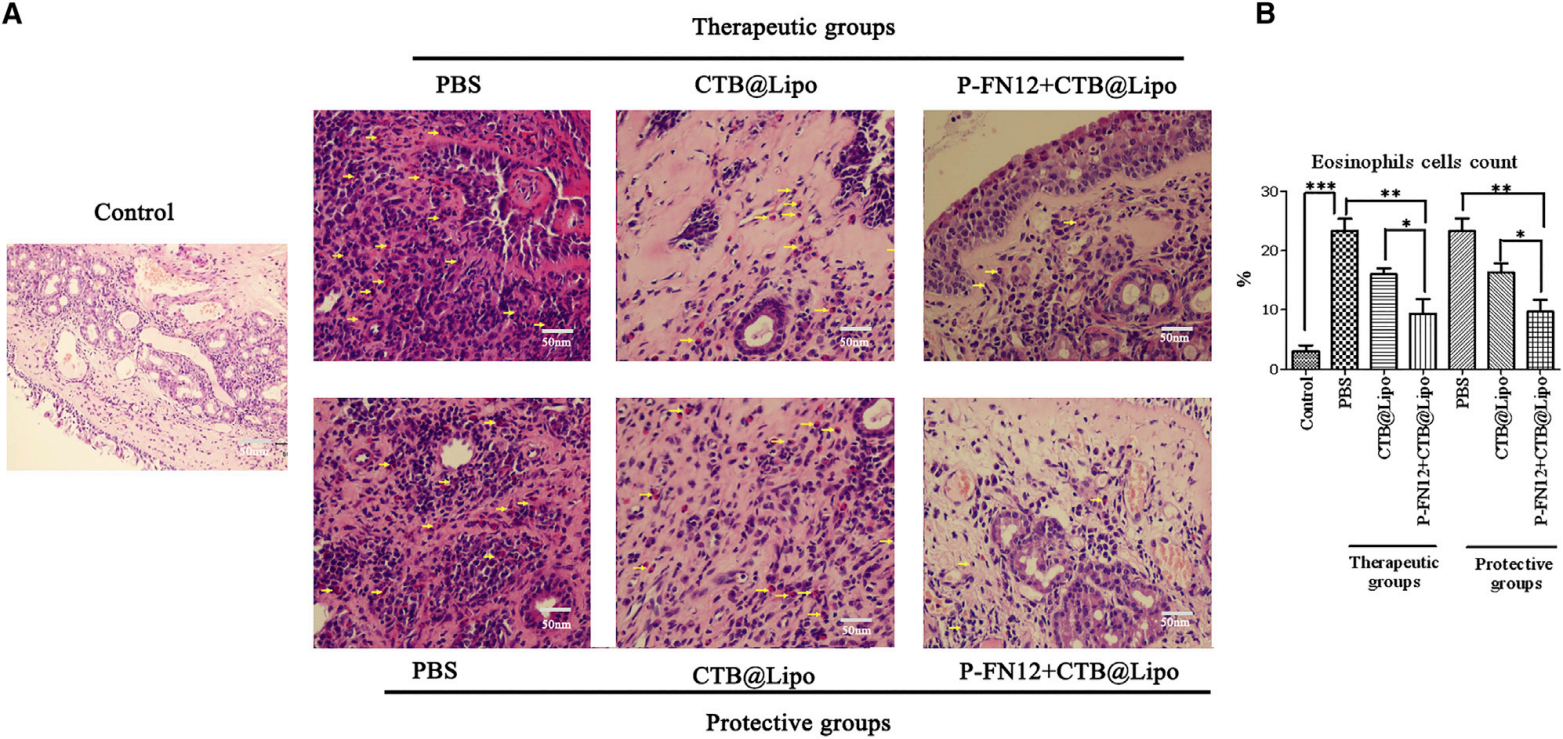
Effects of P-FN12 + CTB@Lipo on the Filtration of Eosinophils into Nasal Mucosa of AR Rats (A) Infiltration of eosinophils (arrows) in the nasal mucosa of rats (n = 5) (H&E, 400×). (B) The proportion of eosinophil cell counts in the nasal mucosa of each group in the field. *p < 0.05; **p < 0.01; ***p < 0.001.

The infiltration of eosinophils in nasal mucosa was shown as Figure 6A. The number of eosinophils was significantly higher in the PBS-treated AR group than in the normal control group (p < 0.001) (Figure 6B). In the P-FN12 + CTB@Lipo-treated AR groups, eosinophil counts were significantly lower than in both the PBS-treated AR group and CTB@Lipo-treated AR group, both in the therapeutic and protective settings (p < 0.01 and p < 0.05). These results indicated that treatment with the P-FN12 + CTB@Lipo vaccine effectively inhibited the infiltration of eosinophils.

## Discussion

Allergic diseases are prevalent worldwide. AR is a disease with symptoms including nasal itching and sneezing. In the present study, AR was induced in rats by intraperitoneal injection and intranasal OVA exposure. The rats in the AR group showed more frequent symptoms of nose rubbing and sneezing compared with the control group. Previous studies have shown that AR is primarily caused by a Th1/Th2 imbalance.^18^, ^19^ Thus, enhancing the Th1 and/or reducing the Th2 immune response to correct the Thl/Th2 imbalance may prevent allergic inflammatory reactions and ease the symptoms of allergic diseases.^20^, ^21^ Allergen immunotherapy (AIT) is the only curative method that can change the immunological response to allergens, leading to modification of the natural course of disease. Histamine is one of the best-characterized pruritogens in humans, and it is known to play a role in pruritus associated with urticaria as well as ocular and nasal allergic reactions. Histamine mediates its effects via four receptors. The most recently discovered histamine receptor, H4R, is a promising target for novel anti-inflammatory agents in several conditions, including asthma and other chronic inflammatory diseases.^13^, ^22^

H4-antihistamines will eventually prove effective and safe in the treatment of allergic disorders, especially in patients with AR.^11^, ^23^ Shiraishi et al.^24^ showed that histamine binds to H4R on basophils, leading to the migration of basophils to the nasal tissue. To our knowledge, however, there have been no previous reports regarding allergens for H4R immunotherapy. To investigate the immune regulatory effects of H4R on Thl/Th2 cell differentiation, we used phage display libraries to predict and analyze possible target epitopes of H4R, and we found that the epitope P-FN12 was bound specifically to anti-H4R antibody (Figure 1). In this study, a P-FN12-based vaccine markedly relieved the symptoms of AR, such as nasal rubbing and sneezing (Figure 3). The symptoms were clearly relieved on day 92 in the therapeutic group and on day 78 in the protective group, indicating that the vaccine would take some time to exert its effect.

**Figure 7:**
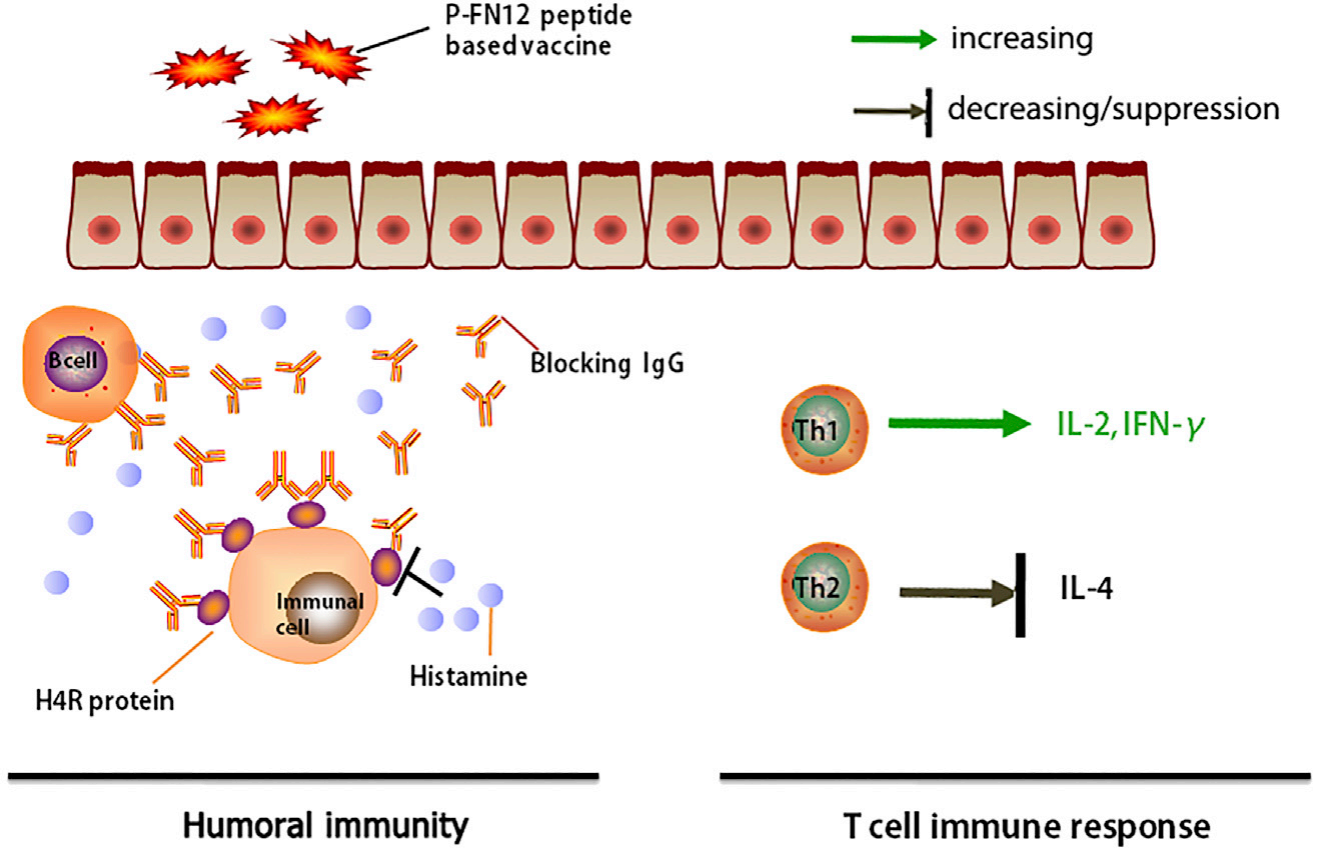
Graphical Depiction of the Immunological Effects of the Formulated P-FN12 Peptide-Containing Vaccine on Immediate Reactions and the Elicited T Cell Immune Response Vaccination induced protective blocking by IgG antibodies. These allergen-specific antibodies thus prevented the binding of histamine to the H4R protein. Another approach can be used to establish an appropriate allergen-specific Th1/Th2 ratio, with the goal of correcting the immune disorder.

Further, it was demonstrated that P-FN12 could induce H4R-specific humoral immunity (Figure 2), which suggests P-FN12 as a potential vaccine target for AR. To test this hypothesis, immediate reaction and T cell immune response were both evaluated (Figure 7). In the immediate reaction, the anti-P-FN12 antibody bound specifically to H4R expressed on immune cells, which blocked the combination of histamine and H4R and, thus, prevented immune cell activation. To examine the T cell immune response, cytokine expression in the serum and nasal mucosa of vaccinated rats and the proportion of Th1 and Th2 cells were evaluated (Figures 4B and 4C). Thus, the P-FN12 + CTB@Lipo vaccine elevated the concentrations of Th1-type cytokines IL-2 and IFN-γ, but it reduced the concentration of the Th2-type cytokine IL-4 in the serum, in both therapeutic and prophylactic settings. Similarly, IL-2 and IFN-γ expression levels were increased in the nasal mucosa, with decreased IL-4 expression levels, suggesting the higher efficacy of the vaccine in reducing the Th2 response. Furthermore, the ratio of Th1:Th2 was increased in PBMCs from vaccinated rats, which suggests that P-FN12 may play a role in affecting the Th1/Th2 imbalance in AR rats. These results further indicate that treatment with P-FN12 + CTB@Lipo effectively corrected the Th1/Th2 imbalance in AR rats. These findings were similar to those reported previously.^25^

The Th2 cytokine IL-4 plays an important role in class-switching (to IgE) and B cell differentiation toward IgE production, whereas the Th1 cytokine IFN-γ inhibits this switch during treatment of allergic disorders.^26^, ^27^ In our present study, the level of OVA-specific IgE was significantly increased upon intraperitoneal OVA sensitization, indicating that the rat model of AR had been appropriately established (Figure 4A). Furthermore, the IgE level decreased after P-FN12 + CTB@Lipo vaccine administration, consistent with the trends in cytokine levels. It also indicated that treatment with P-FN12 + CTB@Lipo vaccine effectively inhibited the infiltration of eosinophils in the nasal mucosa (Figure 6), which suggsted the alleviation of AR symptoms.

To evaluate the toxicity of the vaccine, the livers and kidneys from vaccinated rats were removed and stained with H&E (data not shown). We found no significant differences in the hepatic sinusoid or lobular architecture of the livers or in the morphological structure of the kidneys. There was also no difference in body weight in rats immunized with P-FN12 + CTB@Lipo (p > 0.05). However, further work is required to explore the longevity of P-FN12 + CTB@Lipo immunotherapy; this is a limitation of our study.

In conclusion, a potential critical epitope from H4R, P-FN12, was identified by use of overlapping synthetic peptides and phage display libraries. The P-FN12 peptide-based vaccine P-FN12 + CTB@Lipo alleviated the symptoms of AR model animals. The vaccine elicited a specific antibody against H4R to suppress immune cell activation, and it caused a shift in the pathogenic Th2 response toward the non-pathogenic Th1 response in a rat AR model. Moreover, P-FN12-based peptide vaccine showed little toxicity in rats. Thus, the liposome-adjuvant allergen vaccine was effective against AR, and it may provide a basis for immunotherapy for allergic diseases.

## Materials and Methods

### Materials

Specific pathogen-free inbred healthy Wistar rats (weight: 250–300 g) were obtained from Beijing Huafukang Bioscience (Beijing, China). The following reagents were used: OVA (grade VII; Sigma, St. Louis, MO), aluminum hydroxide hydrate gel (Cosmo Bio, LSL, Tokyo, Japan), cholera toxin B subunit (*CTB*) (United Cell Biotechnology, Shanghai, China), and an H4R antagonist (JNJ7777120, Selleck China).

### Phage Display Library Screening of Anti-H4R Antibodies

96-well microtiter plates were coated with affinity-purified anti-H4R antibody (40 μg/well) in sodium bicarbonate buffer (pH 8.6) and incubated overnight at 4°C. Blocking buffer consisting of 1% (w/v) BSA/Tris-buffered saline (TBS) was added to each well and incubated for 2 hr at room temperature, followed by 1 hr at 37°C using the phage library (total of 2 × 10^11^ phages). The wells were washed six times with TBS and Tween 20 (TBST). Unbound phages were discarded and the wells were washed five times with TBST. Bound phages were eluted by adding elution buffer (0.2 M glycine-HCl in 1 mg/mL BSA [pH 2.2]) to each well, and the plates were gently rocked for 12 min at room temperature. The eluate was neutralized with 1 M Tris-HCl (pH 9.1). The eluate was amplified and precipitated with 20% (w/v) PEG-8000. A second round of panning was conducted using the first round of amplified eluate as the input phages. The entire screening protocol was repeated for a total of three rounds of panning.

After the final round of panning, several phage clones were screened. 96-well microtiter plates were coated with affinity-purified anti-H4R antibody (40 μg/well), and individual phage clones were added separately to each well and incubated for 1 hr, followed by 1 hr of incubation with horseradish peroxidase (HRP)-labeled anti-M13 pIII mcAb (1:2,500 dilution; New England Biolabs, Ipswich, MA). The DNA of the phage clones with high binding affinity toward anti-H4R antibody was extracted, and the nucleotide sequences were determined (GENEWIZ Bioscience Enterprise, Beijing, China). The homology of deduced amino acid sequences (compared to the primary sequence of H4R) was assessed using the BioEdit Sequence Alignment Editor.

### Specificity Evaluation of Active Phage Clones and Synthetic Epitope Using ELISA

The potential epitope P-FN12 (FNKWMDCLSVTH; purity > 95%) was synthesized by Huiyuan Biotechnology (Shanghai, China) using solid-phase peptide synthesis techniques. The specificity was evaluated as described below.

96-well microtiter plates were coated with affinity-purified anti-H4R antibody (1 μg each), anti-CEA antibody, and BSA protein in sodium bicarbonate buffer (pH 8.6) and incubated separately overnight at 4°C. Blocking buffer consisting of 1% (w/v) BSA/TBS was added to each well and incubated for 2 hr at room temperature, followed by 2 hr of incubation at 37°C with purified phage P-FN12 and control phages (titer: 10^10^). The wells were washed six times with PBST. HRP-labeled anti-M13 pIII mcAb was added to each well and incubated for 1 hr at 37°C. The wells were then washed three times with PBST and incubated with 3,3′,5,5′-tetramethylbenzidine (TMB; Amresco, Solon, OH) for 30 min in the dark. A stop solution consisting of 2 M H_2_SO_4_ was added to stop the reaction, and the absorbance at 450 nm (OD_450_) was measured.

### Sensitization of the Rat Model

This study conformed to the Guide for the Care and Use of Laboratory Animals published by the NIH (85-23, revised in 1996). The study protocol was approved by the Animal Ethics Committee of Jilin University.

Basic sensitization was performed by intraperitoneal injection of 1 mL allergen suspension, containing 50 mg OVA and 20 mg Al(OH)_3_ gel, into rats on the first and seventh days. From day 21, 25 μL 5% OVA suspension was administered bilaterally into the nasal cavities of the rats for excitation, a total of seven times a day. From day 27, 25 μL 5% OVA suspension was administered twice a week until day 85. The control groups received sterile 0.9% sodium chloride solution. Each rat was evaluated after the last stimulation according to the evaluation standards. The procedure is shown in Figure 1.

### Vaccine Preparation and Treatment Protocol

The polypeptide and CTB were dissolved in sterile 0.9% sodium chloride solution, and the same volume of liposomes (Lipofectamine; Invitrogen) was added. Incubation was performed overnight at 4°C for full integration, followed by a return to room temperature before use.

**Figure 8:**
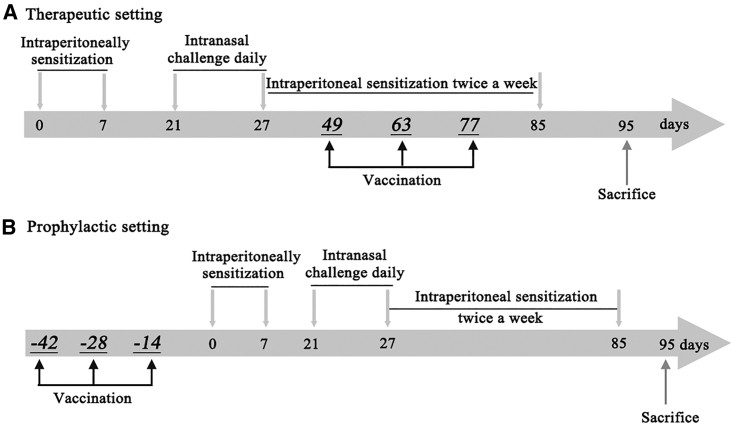
Schematic Representation of the AR Rat Model (A) Therapeutic setting. Rats were sensitized with OVA and Alum on days 0 and 7. All groups except controls received intranasal OVA from days 21 to 85. Rats were sacrificed on day 95. Vaccine was administered on days 49, 63, and 77. (B) Prophylactic setting. Rats were vaccinated every 2 weeks (a total of three vaccinations) before sensitization. The sensitization procedure was the same as that described above. AR, allergic rhinitis; OVA, ovalbumin; Alum, aluminum hydroxide.

Rats were randomly subdivided into several groups of five rats each (n = 5): the control AR group, P-FN12 therapy group, P-FN12 prevention group, control vaccine therapy group, control vaccine prevention group, and H4-antihistamine therapy group. After successfully establishing the model, Wistar rats were immunized with 150 μg synthetic peptide P-FN12 in adjuvant (P-FN12 + CTB@Lipo) via the intraperitoneal route on day 49. Boosters were administered on days 63 and 77 post-immunization (Figure 8A). Rats in the negative control group were injected with sodium chloride solution (100 μL). The prevention groups were vaccinated every 2 weeks, for a total of three times, at the same dose. At 2 weeks after the final vaccination, the rats were sensitized as described previously (Figure 8B). As a positive control, an H4R antagonist (JNJ7777120) was administered via subcutaneous injection on days 49, 56, and 63 at 10 mg/kg in the therapeutic setting.^28^

Wistar rats were anesthetized by intraperitoneal injection of 10% chloral hydrate (0.3–0.35 g/kg), and 5-mL blood samples for assay of IgE and cytokine levels were collected from the heart on day 90. Nasal mucosal samples for western blotting were also collected on day 90. Rat PBMCs to be evaluated via flow cytometry were harvested on day 85 post-immunization, and they were grown for 65 hr in an incubator at 37°C in an atmosphere of 5% carbon dioxide (CO_2_).

### Humoral Immune Response Induced by P-FN12 + CTB@Lipo

Pooled sera (n = 5) were prepared from the whole-blood samples of each group. H4R-specific IgG levels in sera were measured via ELISA. Briefly, 96-well flat-bottomed ELISA plates (Greiner; Sigma-Aldrich, St. Louis, MO) were coated overnight at 4°C with H4R protein (5 μg/mL) diluted in PBS (pH = 7.2). The plates were incubated with blocking buffer (1% BSA in PBS) for 2 hr at 37°C. The pooled sera were diluted 1:50 in dilution buffer (0.5%, v/v, Tween-20 in blocking buffer), added to the plates, and incubated for 2 hr at 37°C. After rinsing with washing buffer, the plates were incubated with biotin-conjugated goat anti-rat IgG (diluted 1:1,000 in 1% BSA/PBS-Tween; Southern Biotechnology, Birmingham, AL) for 2 hr at 37°C. The plates were then washed and incubated with TMB (Amresco) for 30 min in the dark. A stop solution consisting of 2 M H_2_SO_4_ was added to stop the reaction, and the OD_450_ was measured.

### Evaluation of Nasal Symptoms

A rat model of OVA-induced AR was used to evaluate the anti-AR effect of the vaccine. To evaluate the nasal symptoms, rats (n = 5) were placed in an observation cage, and the frequencies of sneezing and nasal rubbing were counted during a 30-min period on days 78 and 92. Scoring criteria were as follows: nasal rubbing gently 1–2 times was scored as 1; intermediate scratching of the nose and face intermittently was scored as 2; and vigorously scratching the nose and face incessantly was scored as 3. Sneezing 1–3 times was scored as 1; 4–10 times was scored as 2; and >11 times was scored as 3. The final score was the sum of the two symptom scores. Assessors were blinded to the treatment protocol.

### Cytokine and IgE Levels in Serum

IL-2, IL-4, and IFN-γ levels in serum were measured using ELISA kits (LUM000; R&D Systems, Minneapolis, MN), according to the manufacturer’s instructions, and OD_450_ was determined on a microplate reader (Biotek, Winooski, VT). Pooled serum samples (n = 5) were diluted 1:2 in deionized water.

Rat sera were evaluated in terms of IgE antibody levels using a specific kit (R&D Systems). In brief, each well of a 96-well flat-bottomed microplate was coated with 0.25 μg OVA diluted in PBS and incubated overnight at 4°C. Each well was then blocked with 100 μL PBS containing 3% (w/v) BSA for 1 hr at 37°C. Serum diluted 1:100 in 1% (v/v) PBS was added to each well (100 μL) and incubated for 2 hr at 37°C. Each well was then incubated with 100 μL specific IgE antibody solution (diluted 1:2,000) for 30 min at room temperature. The enzyme reaction was developed using TMB as substrate, for 10–15 min, and terminated with 2 M H_2_SO_4_. Optical density (OD) at 450 nm was measured with the aid of a microplate reader.

### Cytokine Analysis of Nasal Mucosa Samples

The nasal mucosa was sampled from the inferior turbinate of AR Wistar rats under general anesthesia. Western blotting was performed. Briefly, total proteins were extracted from the isolated nasal mucosa (n = 5) and placed in 100 μL radioimmunoprecipitation analysis (RIPA) lysis buffer. The protein concentration in the supernatants was determined using the bicinchoninic acid (BCA) method. Samples containing 32 μg protein were boiled and subjected to SDS-PAGE in 10% Tris-glycine gels. The separated proteins were electrophoretically transferred onto polyvinylidene fluoride (PVDF) membranes. The membranes were incubated in 5% fat-free skim milk in TBST for 1 hr at room temperature, and then they were incubated with rat anti-rat IFN-γ, IL-2, IL-4, and β-tubulin mcAbs (Santa Cruz Biotechnology) diluted 1:2,000 and stored at 4°C overnight. The membranes were washed and incubated in goat anti-rat IgG antibodies (Invitrogen) for 1 hr. Then, the membranes were washed three times with TBST and visualized using Western Lightning-ECL, Enhanced Chemiluminescence Substrate (NEL100001EA; PerkinElmer, Waltham, MA).

### Flow Cytometric Analysis of Th1/Th2 Cells

Cell-surface staining was performed by incubation of 50 μL PBMC suspension simultaneously with 10 μL phycoerythrin (PE)-conjugated anti-rat CD3 and 10 μL allophycocyanin (APC)-conjugated anti-rat CD4 mAbs (BD Pharmingen, BD Biosciences, San Jose, CA) for 20 min at room temperature in the dark. An aliquot of 1 mL erythrocyte lysate was added to the incubation. The remaining cell suspensions were incubated with 1 mL erythrocyte lysate for 20 min and then centrifuged to remove the supernatant. After permeabilization and fixation with 200 μL Cytofix/Cytoperm, the cells were permeabilized with 0.1% saponin and labeled for 10 min with APC-labeled anti-rat fluorescein isothiocyanate (FITC)-IFN-γ or eFluor660-IL-4 in the dark. Th1 cells (defined as CD3^+^CD4^+^IFN-γ^+^) and Th2 cells (defined as CD3^+^CD4^+^IL-4^+^) were detected by flow cytometry (FACSAria; BD Biosciences), and data were analyzed using CellQuest software.

### Staining of Eosinophils

After deparaffinization of the samples, the nasal tissue specimens were stained with H&E, and the numbers of eosinophils were counted under 400× magnification. Eosinophils were defined morphologically by the presence of 2-lobed nucleus and eosinophilic granules in the cytoplasm. Individuals who were blinded to the animals’ group assignments counted the number of eosinophils under a light microscope.

### Statistical Analysis

At least two independent experiments were carried out for all tests. All data are presented as means ± SDs. Statistical analysis was performed by one-way ANOVA with Dunnett’s test or Student’s unpaired t test. Two-way ANOVA was used in statistical analysis of nasal symptom scores. In all analyses, p < 0.05 was taken to indicate statistical significance.

## Author Contributions

Conception or design of the work: L.L. and Y.W.; Data collection: Y.W., J.S., and H.W.; Data analysis and interpretation: Y.W., L.A., T.L., and L.L.; Drafting the article: Y.W. and L.L.; Critical revision of the article: Y.W. and L.L.; Final approval of the version to be published: Y.W. and L.L.

## Conflicts of Interest

The authors have no conflicts of interest.
